# PRF‐Assisted Regenerative Endodontic Retreatment of an Immature Tooth: A 4‐Year Follow‐Up

**DOI:** 10.1002/ccr3.71306

**Published:** 2025-10-20

**Authors:** Pegah Sarraf, Bardia Goldani, Mehrfam Khoshkhounejad, Fatemeh Hamidzadeh

**Affiliations:** ^1^ Department of Endodontics, School of Dentistry Tehran University of Medical Sciences Tehran Iran; ^2^ Students' Scientific Research Center Tehran University of Medical Sciences Tehran Iran

**Keywords:** regeneration, retreatment, revascularization, revitalization

## Abstract

Regenerative endodontic retreatment can be a viable nonsurgical option for previously treated immature teeth with persistent periapical pathology. With proper case selection, meticulous disinfection, and preservation of stem cell viability, long‐term healing and continued root development can be achieved even in complex retreatment cases.

## 1 Introduction

Regenerative endodontics has been defined as “biologically‐based procedures designed to replace damaged structures, including dentin and root structures, as well as cells of the pulp‐dentin complex” [1]. Regenerative Endodontic Treatments (RETs) used to be done in necrotic cases [2, 3]. Although recent studies have claimed that the newly generated tissues are not the former ones [4], these treatments are still of great value because of their potential to contribute to root development and root structure reinforcement [5]. There is evidence indicating the higher fracture resistance of the immature teeth that underwent RETs [5]. The latter may indicate a higher survival rate of RET cases in comparison with traditional treatment cases [6].

Root canal treatment may fail in approximately 15%‐25% of cases [7]. The success of nonsurgical root canal treatment is defined as the absence of pain, swelling, and other symptoms, no sinus tract, no loss of function, and radiographic evidence of normal periodontal ligament. Another good indicator of a favorable prognosis is the resolution of apical radiolucency and evidence of a re‐establishment of the lamina dura [8]. Non‐surgical endodontic retreatment has long been recommended in such cases to remove the infection source and facilitate healing [9].

Among scaffolds proposed for regenerative endodontics, autologous platelet concentrates (APCs) such as PRP, PRF, and their modifications have gained attention in medicine and dentistry. Platelet‐rich fibrin (PRF) is a second‐generation platelet concentrate derived entirely from the patient's own blood, making it biocompatible and eliminating the risk of immune reactions or disease transmission. Its advantages include its autologous origin, simple preparation, cost‐effectiveness, and versatility in clinical applications [10]. PRF is rich in platelets, leukocytes, cytokines, circulating stem cells, and macrophages, and contains various growth factors, including platelet‐derived growth factor (PDGF), transforming growth factor‐β1 (TGF‐β1), insulin‐like growth factor (IGF), and vascular endothelial growth factor (VEGF). These biologically active components play essential roles in stimulating angiogenesis, modulating inflammation, promoting cell proliferation, and enhancing tissue repair. In endodontics, PRF has been utilized in numerous scenarios where tissue healing and regeneration are essential, including the management of endodontic–periodontal lesions, sealing of root perforations, vital pulp therapy, and as a scaffold in regenerative endodontic protocols [11]. Its ability to provide a three‐dimensional fibrin matrix not only supports cellular migration and proliferation but also ensures a sustained release of growth factors over time, making it an ideal adjunct in regenerative dentistry [10]. Advanced PRF (A‐PRF+) protocols further optimize fibrin architecture and growth factor release, making it a promising scaffold for regenerative endodontics [12].

Multiple formulations of PRF have been developed to optimize its clinical performance. These include leukocyte‐rich PRF (L‐PRF), injectable PRF (i‐PRF), and advanced PRF (A‐PRF). Advanced PRF (A‐PRF) is a modified form obtained by slightly increasing centrifugation time and lowering speed, resulting in improved regenerative potential compared with standard PRF. A‐PRF has been widely applied in periodontal regeneration and implant surgery, with promising results also reported in endodontic surgery [13]. Compared with L‐PRF, A‐PRF contains a higher total viable cell population, including an increased number of immune cells. This enhanced cellular content is believed to influence macrophage differentiation and maturation, which play a crucial role in bone and soft tissue healing through macrophage‐mediated release of growth factors. A‐PRF additionally provides a three‐dimensional scaffold and reservoir for growth factors, and studies suggest it may contribute to increased root thickness in regenerative endodontic procedures [14].

Advances in technology have evolved root canal retreatment quality and outcomes [15, 16]. Recruitment of a regenerative approach in the retreatment of immature teeth is a new approach aiming to give a chance to immature teeth to develop after conventional treatments were not successful. Recently, some case reports have recruited a regenerative approach in such cases, and the outcomes indicated “success” [17, 18]. The American Association of Endodontists (AAE) clinical considerations for regenerative endodontic procedures define success by three measures [19]: Primary goal (essential): The elimination of symptoms and the evidence of bony healing; Secondary goal (desirable): Increased root wall thickness and/or increased root length; Tertiary goal: positive response to vitality testing [20]. In the present case, we recruited a regenerative endodontic protocol (REP) by means of which we would have not only an asymptomatic tooth, healing of the periapical lesion, and the bone surrounding the perforation site, but also restore tooth vitality.

## 2 Case History/Examination

**FIGURE 1:**
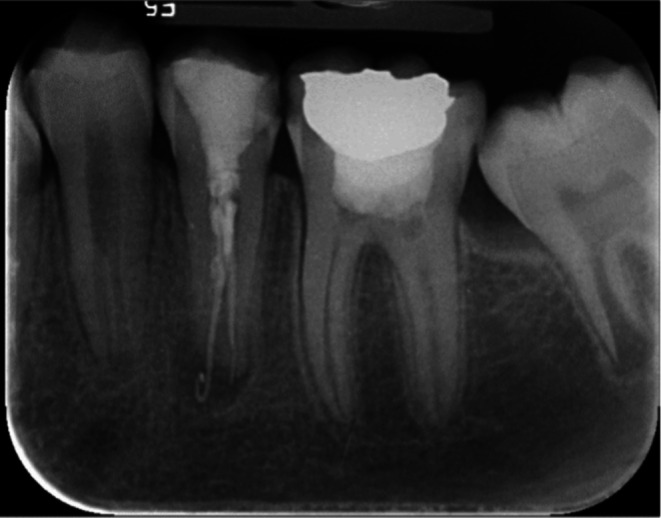
Initial Radiograph with poor‐quality previous root canal filling and periapical lesion.

A 14‐year‐old girl was referred due to pain on chewing and a history of acute apical abscess on tooth No. 20 to the Department of Endodontics, Faculty of Dentistry, Tehran University of Medical Sciences, on January 4, 2021. The tooth had undergone root canal therapy and direct composite restoration about a year ago. It was sensitive to percussion and did not respond to sensibility tests. Neither a sinus tract nor any swelling was detected at the first visit. The periapical view revealed a poor‐quality previous root canal treatment associated with a periapical lesion and, probably, an apical perforation (Figure 1). Thus, CBCT was ordered. CBCT also revealed an apical lesion and root perforation in the apical third of the root with bone resorption adjacent to the perforation site. Informed consent was taken from the patients' parents, and a regenerative treatment modality was considered.

**FIGURE 2:**
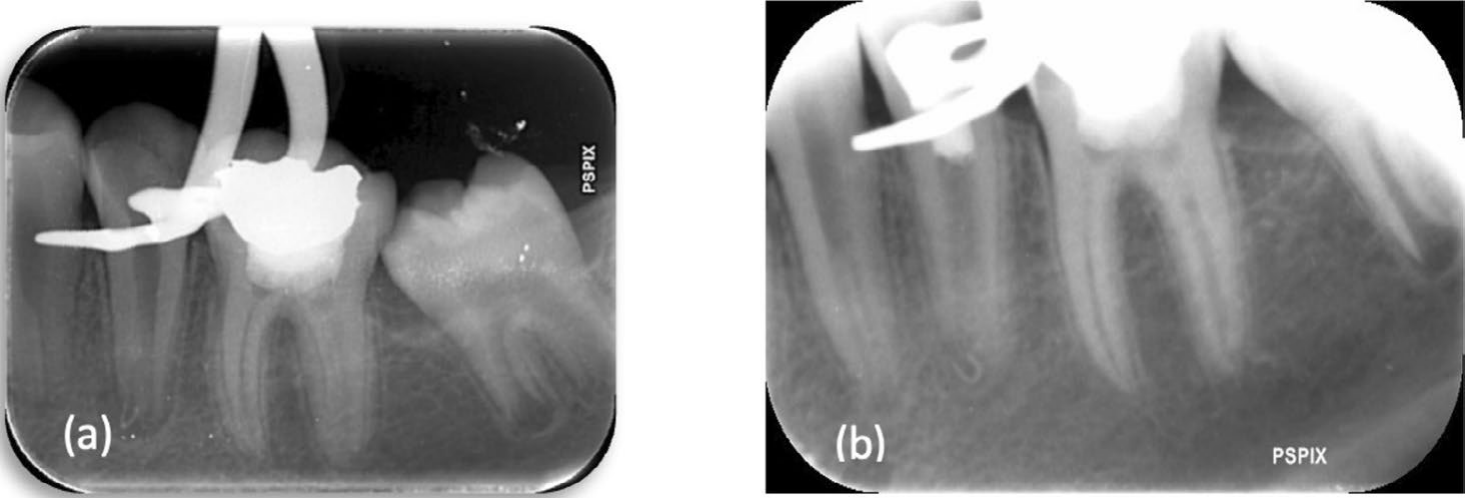
(a) After gutta‐percha removal; (b) Post‐operative radiography.

One cartridge of local anesthetic solution (2% lidocaine + 1:80000 epinephrine) was injected using the incisive nerve block technique. Under rubber dam isolation, restoration, and recurrent caries were removed. An access cavity was prepared. Gutta‐percha was removed under magnification; however, a piece of gutta‐percha was pushed out of the root, which could not possibly be removed (Figure 2). Working length was determined using an electronic length measuring device (Root ZX II, Morita, Japan). Working length was verified using a periapical view radiograph. Canal preparation was done using the SP1 rotary system (SP1, Fanta Dental, China) up to F3. The canal was irrigated with 10 mL of 1.5% sodium hypochlorite and 17% EDTA (Morvabon, Iran) using a 30‐gauge, close‐ended, double‐sided, vented needle. Then, the canal was dried and a mixture of ciprofloxacin and metronidazole based on the AAE protocol [20] was applied in the canal using a #25 lentulo spiral (Dentsply Maillefer, Ballaigues, Switzerland). Reinforced ZOE (Zoliran, Golchadent, Iran) was provided as the temporary coronal seal.

Two weeks later, at the second visit, the patient reported no pain on percussion or on chewing. She also did not report any swelling or abscess formation since the last visit. After local anesthetic solution (3% Mepivacaine) injection using the incisive nerve block technique, the temporary restoration was removed under rubber dam isolation. The medicament was removed via chemo‐mechanical debridement. The canal was irrigated with 17% EDTA according to AAE clinical consideration for regeneration [20]. Then the canal was dried. Dentin bonding agent (3 M Adper Single Bond, 3 M, USA) was applied in the pulp chamber to reduce discoloration.

A 10 mL venous blood sample was collected from the patient's right cubital vein using a butterfly needle under aseptic conditions by a qualified nurse. The sample was immediately centrifuged at 1300 rpm (≈200 g) for 8 min. Centrifugation yielded three layers: platelet‐poor plasma (supernatant), a middle PRF fibrin clot, and red blood cells (RBCs) at the bottom. The PRF clot was aseptically retrieved with sterile tweezers, separated from the RBC layer, and trimmed as necessary. The clot was then placed in a PRF box to achieve gentle compression, producing a fibrin membrane, which was subsequently cut into the required size for application.

**FIGURE 3:**
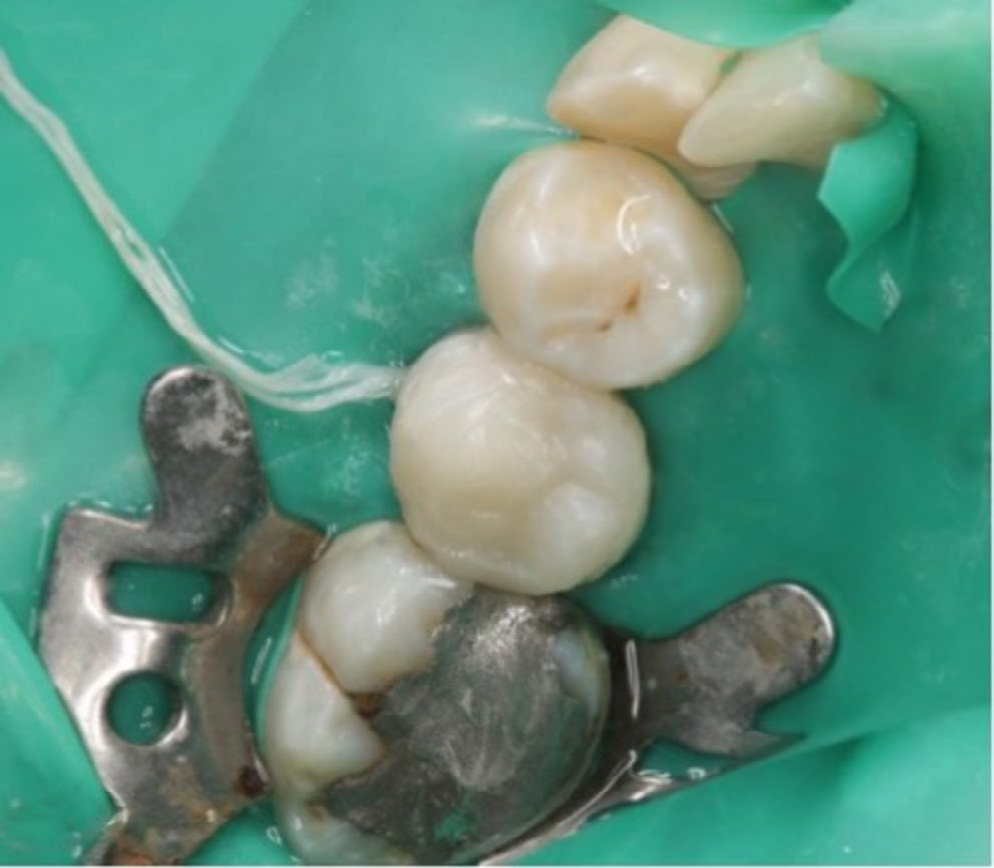
Definitive restoration.

After preparation of the PRF plug, bleeding was induced by over‐instrumentation of a #10 K‐file (Mani, Japan). While bleeding was controlled about 5 mm under the cementoenamel junction (CEJ), the prepared PRF plug was applied in the canal. A 3‐mm‐thick RetroMTA (BioMTA, Korea, Seoul) barrier was applied just apical to the CEJ. The tooth was temporarily restored by resin‐modified glass ionomer (GC Fuji IX, GC Co., Tokyo, Japan) (Figure 2). After 1 week, MTA setting was checked, and the patient was referred to the department of restorative dentistry. Composite build‐up restoration was conducted under rubber dam isolation (Figure 3).

## 3 Conclusion and Results

**FIGURE 4:**
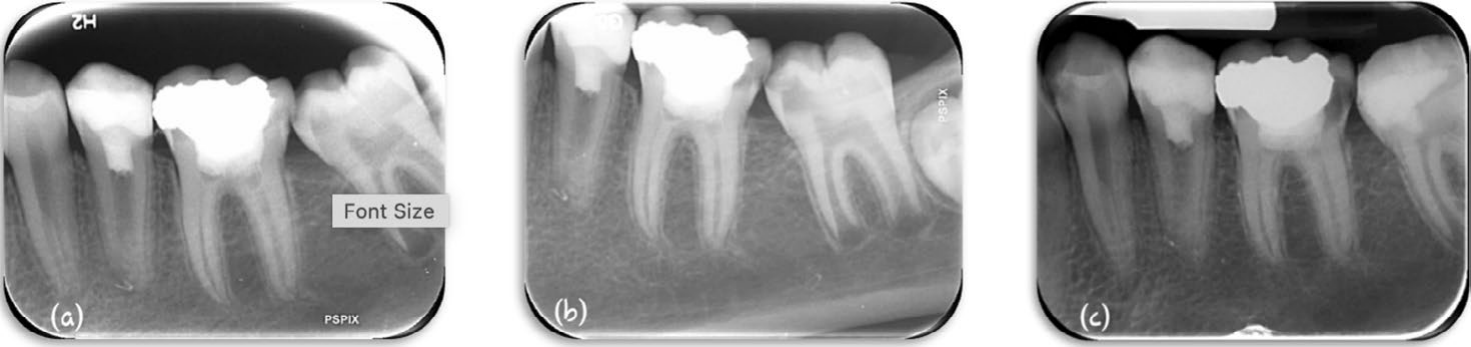
(a) Six‐month, (b) 12‐month, and (c) 18‐month follow‐ups.

The patient was followed at 6‐, 12‐, and 18‐month intervals. In each follow‐up session, a periapical view radiograph was taken (Figure 4), and the tooth was examined to check if any swellings, sinus tracts, pain on percussion, or periodontal pockets were present. No signs of recurrent, persistent, or emerging infection were observed at follow‐up sessions. The tooth did not respond to sensibility tests at follow‐up sessions.

**FIGURE 5:**
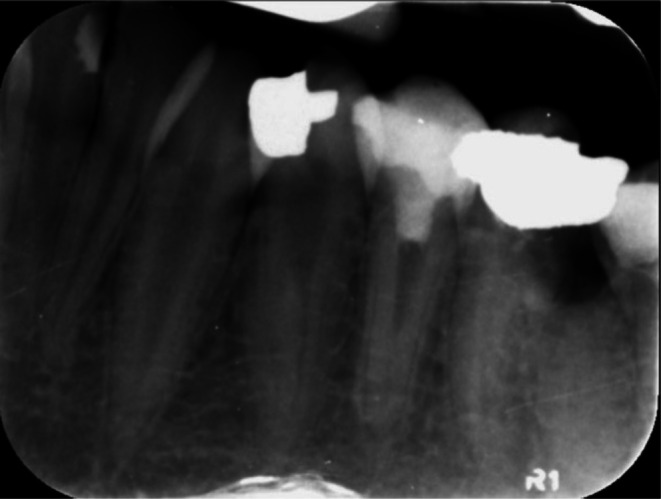
Four‐year follow up.

After 4 years, the tooth remained asymptomatic but still didn't respond to sensibility tests. Radiographic examination revealed complete healing of the periapical lesion, apical closure, and normal periapical lamina dura. Increased radicular dentin thickness and longitudinal root development were indisputable in the 4‐year follow‐up (Figure 5).

## 4 Discussion

The idea of regenerating pulpal tissue was primarily proposed as a treatment option for vital immature teeth [21]; then it was extended to mature/immature necrotic cases [20, 21]. Although it is not generally accepted to take a regenerative approach in retreatment of immature cases, the idea does not seem irrational [17, 18].

The disinfection of root canals in teeth with necrotic pulp and open apices undergoing RET is complicated by several factors. Bacterial penetration in dentinal tubules of young patients is enhanced by their wider diameter compared with that of older patients. The canal space is frequently larger, thus providing a larger surface area for microbial biofilms and interfering with adequate cleaning and shaping [22].

In the present case, previous treatment was done about a year ago, and the negative point of this is the probable mature biofilm, which is more difficult to remove completely, and this long‐lasting situation may compromise the RET outcome. As Nosrat et al. revealed, teeth with a history of pulp necrosis for an extended period of time (more than 6 months) showed poor radiographic root development after RET [2, 17]. Effective disinfection in regenerative endodontic treatment is essential to establish a favorable healing environment by eradicating bacteria; however, preserving stem cell viability is equally important. Therefore, clinicians must balance the antimicrobial potency of irrigants and medicaments with their potential cytotoxic effects on stem cells [22]. Key challenges in this case were removing previous root filling material from the root canal system and achieving optimal cleaning and shaping without causing iatrogenic damage.

Studies have shown a significant contrast in the microbial phenotypes of primary and secondary endodontic infections. Primary infections are typically polymicrobial, with a predominance of anaerobic bacteria, whereas secondary infections are dominated by Gram‐positive species, particularly *Enterococcus faecalis* [23]. *E. faecalis* is known for its resistance to commonly used intracanal disinfectants, its ability to survive in nutrient‐deprived environments, and its capability to form resilient biofilms, all of which contribute to persistent apical periodontitis [23, 24]. The presence of resistant and persistent species made the disinfection process in this case more challenging.

The choice of scaffold is critical for the success of RET. While induction of a blood clot has traditionally been the simplest method, its limitations include variability in clot formation and growth factor content. Platelet concentrates such as PRP and PRF have been introduced as more reliable alternatives. PRP provides a high concentration of growth factors but requires anticoagulants and bovine thrombin, raising biocompatibility concerns. In contrast, PRF is obtained through a simpler, anticoagulant‐free process and offers a fibrin network that allows sustained release of growth factors, supporting angiogenesis and tissue repair [11]. Recent studies have reported enhanced outcomes with advanced PRF (A‐PRF+), which contains higher leukocyte content and improved fibrin architecture, enabling prolonged release of vascular endothelial growth factor (VEGF), platelet‐derived growth factor (PDGF), and transforming growth factor beta (TGF‐β) [13]. These properties likely contributed to the successful periapical healing and continued root maturation observed in this case.

In the present case, A‐PRF^+^ was utilized as a scaffold. A recent in vitro study assessed the osteogenesis potential of three PRF preparation protocols, including L‐PRF, i‐PRF, and A‐PRF^+^, and concluded that A‐PRF^+^ has the highest potential in mineralization [25]. Although favorable outcomes of regenerative treatments are multifactorial, the selection of a suitable scaffold may significantly contribute to successful tissue regeneration, esp. in the present case.

Gutta‐percha removal is critical in the management of failed endodontic treatment. The use of solvents in endodontic retreatment has been offered as a viable approach to increase the cleaning efficacy and success rate of retreatment operations by easing the removal of remaining filling and debris from the root canal [26]. The use of solvents in cases like this has more challenges because solvents, especially chloroform, have been shown to be highly toxic in several studies [27, 28]. The effect of these solvents on the proliferation, attachment, and differentiation of stem cells and on the dentinal walls has not been studied, and the risk of extrusion of previous material to the periapical area is present.

We assessed our clinical/radiographic success based on AAE Clinical Considerations for REPs [24]. Elimination of the signs and symptoms, followed by evidence of bone healing, indicated that the primary aim was achieved. Apical closure, as the secondary aim, assessed by periapical radiographic view, was gained to some extent. Although the final or desirable goal, which is tooth response to sensibility tests, was not achieved, bone healing adjacent to the perforation site, despite foreign body remnants, was evident. To the best of our knowledge, the latter is reported for the first time following regenerative endodontic treatment.

A noteworthy outcome of this case was the lack of responsiveness to pulp vitality testing throughout the follow‐up period, despite clear clinical and radiographic evidence of healing. This phenomenon has been frequently reported in regenerative endodontics. Several mechanisms may account for the discrepancy between radiographic healing and sensibility recovery. First, vascular regeneration is often more predictable than neural regeneration, and sensory nerve ingrowth may lag behind angiogenesis [29]. Second, the inherent limitations of sensibility tests, which rely on functional sensory nerve response rather than true vitality, may contribute to false‐negative outcomes [30]. Thus, the persistent negative vitality test observed in this case does not necessarily indicate treatment failure but reflects the biological variability of tissue repair.

This case highlights how regenerative endodontic retreatment can offer a promising nonsurgical option for immature teeth with persistent periapical pathology. Initially, the need to remove existing obturation material while ensuring thorough disinfection—without compromising stem cell viability—cast some doubt on the prognosis. Yet, the favorable outcomes observed over a four‐year follow‐up demonstrate that these concerns can be overcome. Given that this is a single‐case report, however, our observations should be viewed cautiously. Larger, controlled studies and extended clinical series are essential to refine protocols, select optimal materials, and identify the factors that predict success in similar retreatment cases. Clinicians exploring regenerative methods for previously treated immature teeth must carefully assess each patient's unique circumstances, commit to meticulous follow‐up, and stay alert for potential complications, all while appreciating the potential of RET to enhance long‐term tooth survival and function.

The strengths of this case include the long‐term follow‐up (4 years), adherence to AAE protocols, and the novel use of A‐PRF+. Limitations include the inherent restriction of a single‐case design, the inability to generalize outcomes, and persistent negative vitality testing.

## Author Contributions

**Pegah Sarraf:** conceptualization, supervision, writing – review and editing. **Bardia Goldani:** methodology, visualization, writing – original draft, writing – review and editing. **Mehrfam Khoshkhounejad:** conceptualization, writing – review and editing. **Fatemeh Hamidzadeh:** conceptualization, investigation, methodology, project administration, supervision, validation, visualization, writing – original draft, writing – review and editing.

## Consent

Written informed consent was obtained from parents to publish this case report in accordance with the journal's patient consent policy.

## Conflicts of Interest

The authors declare no conflicts of interest.

## Data Availability

The data that supports the findings of this study are available on request from the corresponding author.
